# Community Priorities for Outcomes Targeted During Professional Supports for Autistic Children and their Families

**DOI:** 10.1007/s10803-024-06333-2

**Published:** 2024-04-20

**Authors:** Rhylee Sulek, Chris Edwards, Ruth Monk, Lee Patrick, Sarah Pillar, Hannah Waddington

**Affiliations:** 1 Menzies Health Institute Queensland, Griffith University, Parklands Dr Southport, QLD, 4222 Gold Coast, Australia; 2 Telethon Kids Institute, Perth, Australia; 3 Autism Spectrum Australia, Chatswood, Australia; 4 Autism New Zealand, Lower Hutt, New Zealand; 5 School of Medical Sciences, University of Auckland, Auckland, New Zealand; 6 Faculty of Education, Te Herenga Waka -Victoria University of Wellington, Wellington, New Zealand

**Keywords:** Autism, Outcomes, Professional supports, Priority, Community perspectives

## Abstract

**Purpose:**

Professional supports play an important role in aiding autistic children’s learning, participation, and overall wellbeing. Yet, limited research exists on stakeholders’ perspectives and preferences regarding targeted outcomes for children undergoing support facilitated by professionals. This study investigated stakeholder views on the priority and appropriateness of outcomes intentionally targeted during the provision of supports to autistic children.

**Method:**

A survey of 181 participants (including 72 autistic adults, 85 parents, and 69 professionals) from Australia and New Zealand was conducted. Participants rated the appropriateness and priority of 47 potential child and parent outcomes within the context of support.

**Results:**

The highest priority outcome was improving child mental wellbeing, with the lowest being reducing sensory seeking or avoidant behaviours. Priority ratings for certain outcomes differed based on the child’s age. Over half of the participants rated *reducing sensory seeking/avoidant behaviours* and *reducing focused interests* as inappropriate outcomes of supports. Further, variations in the appropriateness of outcomes differed among participant groups.

**Conclusion:**

Reflecting the growing acceptance of neurodiversity-affirming practices, these results underscore support for targeting outcomes that are meaningful to the autistic and autism communities, with less emphasis on those which reflect neurotypical behavioural standards.

**Supplementary Information:**

The online version contains supplementary material available at 10.1007/s10803-024-06333-2.

Supports (which may also be referred to as therapies or interventions) provided during childhood can assist in laying the foundation for positive learning, participation, and wellbeing outcomes for all children, and may be particularly important for children who are, or may be, autistic (Cioni et al., 2016; Trembath et al., 2022). Traditional models of support for autistic children were underpinned by the medical model of disability, where autism is approached as a condition to be addressed by targeting a child’s fundamental ‘impairments’ (Gutkin, 2012; Sheridan & Gutkin, 2000). An approach which has been widely criticised for its deficit-based approaches seeking to change the nature and characteristics of the autistic child (Kapp et al., 2013). In contrast, a social model perspective (Oliver, 2013) posits that the disability associated with autism arises from the child’s environment, leading supports to focus on specific changes in the child’s surroundings and broader societal shifts. A biopsychosocial model (World Health Organization, 2001) integrates the medical and social approaches, recognising the interaction between aspects of children’s development and the environment, and suggests supports should address both for enhanced participation. There is a growing shift in the past two decades towards strengths-based approaches to providing professional supports in line with the neurodiversity movement (Pellicano & den Houting, 2022). Neurodiversity-affirming approaches highlight the value of natural human variation, emphasising the need for individualised support and increased education and training of people who support autistic children, in addition to creating more autism-friendly environments (Botha et al., 2022; den Houting, 2019; Kapp, 2020). These evolving understandings of autism and the role of professional supports are likely to lead to a shift in the outcomes which autistic children and families consider to be both appropriate and important.

Clarifying the intended outcome of any planned support is an important step during support provision. In the extant literature, the types of outcomes frequently targeted tend to be limited in scope. In a research context, primary outcomes studied in response to the provision of early supports have largely been limited to core autism characteristics or related child skills (e.g., cognition or language acquisition) with far less attention paid to personal or social functioning outcomes (e.g., child and family participation and wellbeing) (see Trembath et al., 2022 for a recent review). Given targeting a reduction in autism characteristics is unlikely to align with more contemporary, neurodiversity-affirming views of support provision (Leadbitter et al., 2021), it is essential to determine the priority of support for various intended outcomes in the autistic and autism communities. This seems particularly salient as these may differ from those of researchers and funders (Benevides & Cassidy, 2020; Emerson et al., 2023; Pellicano et al., 2014).

For example, Emerson et al. (2023) found a clear mismatch between the types of projects funded by research schemes and those areas considered to be most important to the autistic and autism communities in New Zealand. Specifically, participants were dissatisfied with the proportion of funding allocated to biologically based research, with a preference for research which demonstrates real world change in the lives of autistic people and their families. Roche et al. (2021) in their synthesis of research examining stakeholder perspectives of key research priorities, similarly, found that research prioritising real world, translatable impact for autistic individuals was often prioritised over biological research. Among the most prioritised research priorities were those which targeted the physical and mental health and wellbeing and learning skills (development and cognition) of autistic individuals.

Recent work by Waddington et al. (2024) investigated the perspectives of the autistic and autism communities in Australia and New Zealand towards the appropriateness and importance of early support goals for children. When asked to consider a hypothetical three year old autistic child in providing their responses, the study found that across all participants, the highest priority support goals were improving child quality of life, reducing harmful behaviours, and targeting adult support for autistic children. Further, they highlighted that, while still considered appropriate goals by some participants, the lowest priority goals were those which focused on changing the child’s autistic characteristics, play, and academic skills, with these goals rated significantly lower in priority by autistic adults (compared to practitioners and parents). This highlights a trend towards supporting more neurodiversity-affirming goals across the autistic and autism communities. Goals can be conceptualised as the incremental steps or aims that may be worked towards during the provision of early supports to achieve a broader outcome. When understanding community perspectives, in light of changing conceptualisations around disability and early support provision, it is important to not only understand the social significance of the discrete goals that might be identified but also to understand the importance of different outcomes of supports might be (Wolf, 1978).

As previously highlighted, research on targeted outcomes of support has been limited in scope (Trembath et al., 2022) with no research, to the authors knowledge, examining community preferences. The present study builds on the work of Waddington et al. (2024), by surveying autistic adults, parents of autistic children, and practitioners living in Australia and New Zealand regarding their views on the appropriateness and priority of both child and parent outcomes that might be targeted during early autism support. We also examined whether appropriateness and priority of outcomes differed across age groups. Consistent with [removed], we also examined current perspectives on early supports and their preferred model of disability to determine whether appropriateness and priority ratings were influenced by participant (a) identity group (autistic adult, parent, or professional); (b) perspectives on the appropriateness of providing early support; and (c) preferred model of disability.

Drawing on the recent findings of Waddington et al. (2024), it was hypothesised that autistic participants would be more likely to express caution around the appropriateness of all types of early supports. Further, autistic adults were hypothesised to align with the social model of disability, with practitioners more likely to align with the biopsychosocial model of disability. Given the exploratory nature, no hypotheses were made for differences in perceived appropriateness and priority of outcomes based on child age. We also anticipated that autistic participants would be more likely to rate outcomes which focus on reducing autism characteristics and changing a child’s social and play skills to be more “neurotypical” as inappropriate intended outcomes of professional supports. Finally, it was hypothesised that certain participant characteristics (i.e., participant identity, perspectives on early supports, and preferred model of disability) would predict priority ratings of both child and family outcomes.

## Method

### Participants

The study was conducted with the approval from the first author’s research institute (HREC 2023/207). Individuals were eligible to participate in the survey if they were living in Australia or New Zealand and were: (a) an autistic individual aged 16 or over, (b) a parent/caregiver of an autistic child aged 18 years and under, and/or (c) a practitioner providing supports to autistic children aged 18 years and under. Participants were recruited via several mechanisms including: survey flyers shared, with permission, in social media groups whose membership represented our target stakeholders (i.e., autistic individuals, practitioners, parents of autistic children); recruitment calls by autism specific organisations to their members via social media or mailing lists; distribution of survey flyers and recruitment emails via the study authors’ professional networks and personal social media pages (e.g., LinkedIn, X, Facebook). The survey was accessed online, with potential participants first reading information about the study and requirements, before choosing whether to consent to participate. As participants under the age of 18 were eligible to complete the survey, these individuals were encouraged to read through the information provided with a parent or caregiver and discuss their participation before providing their consent.

### Measures

A survey was developed to answer the research questions. The survey was divided into five sections: (1) participant demographics; (2) views on providing early supports; and (3–5) appropriateness and priority level of outcomes targeted for: (a) autistic children aged 0–5 years, (b) autistic children aged 6–12 years, and (c) parents of autistic children.

#### Section 1: Demographics

Section one collected information about participants, including screening questions to determine their eligibility. Participants were asked whether they identified as autistic, a parent/caregiver, or a practitioner, and were able to select more than one identity where relevant (i.e., a parent might also be autistic themselves). After selecting all relevant identities, participants were then asked to self-select a primary identity for the purpose of completing the demographic information (i.e., to reduce survey length. Demographic information is presented according to primary identity selected. All participants provided information on their gender, ethnicity, and highest level of formal education. Further information collected from autistic adults included their age, diagnostic status and any co-occurring conditions, and whether they accessed professional supports, and the type of supports accessed, during childhood. Parents provided details for their child(ren), including their relationship to the child(ren), details regarding their child’s diagnosis and co-occurring conditions, and whether their child had or was accessing professional supports, and the type of supports accessed. Practitioners were asked to provide information about their current role, time spent in this role, and the types of settings they worked in.

#### Section 2: Early Supports and Models of Disability

In section two, participants indicated if they thought it was appropriate to provide early supports to autistic children and selected their preferred model of disability/support based on brief descriptions that broadly aligned with medical, social, biopsychosocial, or strengths and challenges models of disability/support. These descriptions were modelled off those used in [removed] and further refined by the research team.

#### Sections 3–5: Appropriateness and Priority of Child and Parent Outcomes

Section three outlined 20 child outcomes that might be intentionally targeted for autistic children aged 0–5 years when receiving professional supports. Section four outlined 21 child outcomes (20 overlapped with those in section three) for autistic children aged 6–12 years. Note that these age groups represent early childhood/preschool (0–5 years) and primary school (6–12 years) age cut offs typically observed in Australia and New Zealand. Section five outlined six parent/caregiver outcomes. For sections three, four and five, participants indicated whether the outlined outcome was an appropriate target of supports. If selected as appropriate, participants rated its priority on a 4-point Likert-type scale, from not at all a priority (1) to a high priority (4). Participants were also able to indicate if they had no opinion/were unsure and were given opportunities to explain their responses in open text responses. In responding to questions in sections two to five, all participants completed these once only, regardless of whether they identified with one than one perspective/identity. A copy of the survey, which includes outcome descriptions, is available in the supplemental file (S4). Participants were also able to provide their contact information in a secondary survey to go in the draw to win a gift-voucher and indicate if they would like to be contacted in the future regarding the study outcomes.

The survey was initially drafted by the first and last authors. The target outcomes identified in sections three to five were based on their knowledge of early autism supports, published literature (Trembath et al., 2022, 2023) and previous work by Waddington et al. (2024). The research team reviewed the draft then met to discuss the format and content, with a particular focus on the target outcomes and definitions. All members of the research team then provided written feedback on the draft and the first author made changes as necessary. Further drafts and revisions were shared via email until consensus was reached. The penultimate draft of the online survey was piloted by an autistic adult, an autistic young person (with their assent and consent from their parent), a parent, and an allied health practitioner. These individuals completed the survey and provided written feedback to the first author about the content and the online platform’s functionality/accessibility. Minor changes were made following piloting, with the final survey approved by all team members.

### Data Management and Analysis

All survey data were collected and managed using the REDCap electronic data capture tools hosted at [removed], a secure, web-based software platform designed to support data capture for research studies (Harris et al., 2009, 2019). A total of 273 responses had been partially or entirely completed. Only participants who (a) met the inclusion criteria and (b) responded to at least one question in the section two, were included in initial analysis (*n* = 201). Additional data screening was also undertaken using a combination of methods to identify inauthentic and/or ‘bot’ responses to the survey. This included a review of patterns of responding, time taken to complete the survey, checking of email addresses provided for the gift-card draw, and cross referencing of time stamps. This led to the exclusion of additional records (*n* = 20) from the dataset. Participant responses for each outcome in sections three to five were coded dichotomously to indicate whether the outcome was considered appropriate or not. They were also coded to create priority scores, with ratings of both ‘not an appropriate goal’ and ‘an appropriate goal but not a priority’ coded the lowest priority (1) (see Waddington et al., 2024). Responses where participants indicated they had no opinion/were unsure were coded as missing for analysis.

All analyses were conducted in IBM SPSS Statistics (IBM Corp, 2022). Descriptive statistics were calculated for participant demographic characteristics, perspectives on early supports, and priority level of child and parent/caregiver outcomes. As multiple statistical analyses were planned, consideration was given to the need to balance the risk of familywise error with the risk of Type 2 errors. A Bonferroni adjustment was considered too conservative, given the number of outcome ratings included (47 in total). An *a priori* decision was therefore made to set the *p* value at 0.01 for all analyses which included outcome variables (Perneger, 1998). The specific analysis approach for each research question is described below and is closely aligned with Waddington et al. (2024).

Before answering our key research questions, we investigated the relationship between participant identity and key variables such as perspectives on early supports and preferred model of disability. A series of Chi-square analyses with post-hoc Bonferroni adjusted pairwise comparisons were conducted to determine the association between participant group and perspectives on early supports and preferred model of disability. The ‘not appropriate’ category was excluded from the early supports analysis as there were too few responses. Similarly, we combined responses which selected the medical model and ‘other’ categories in our model of disability analysis.

#### Do Participant Perspectives on Appropriateness and Priority of Child Outcomes Differ According to Child Age?

To determine whether the *appropriateness* of child outcomes differed for younger versus older children, an exploratory analysis using McNemar’s test was conducted. To determine whether the *priority ratings* of outcomes differed for younger versus older children, an exploratory analysis using Wilcoxon signed-rank tests was conducted. The ‘self-advocacy’ outcome was excluded from both analyses as this only pertained to children aged 6–12 years.

#### Do Perspectives on Appropriateness and Priority of Child and Family Outcomes Differ According to Participant Perspectives?

To determine whether there was an association between perspectives regarding the appropriateness of outcomes and participant group, we conducted a series of Chi-square analyses with post-hoc adjusted pairwise comparisons. Variables without significant differences between children aged 0–5 and 6–12 years were collapsed for analysis. Further, only outcomes where expected cell counts were greater than five were included in analysis. A series of multiple regressions were conducted to determine whether participant characteristics, specifically participant identity, perspectives on early supports, and models of disability, predicted priority ratings of child and family outcomes. Variables without significant differences in priority ratings between children aged 0–5 and 6–12 years were averaged for analysis. Further, only outcomes with sufficient variability were included in regression analysis, that is, outcomes with ≥ 75% of responses in any one response category were excluded.

### Community Involvement

Three autistic individuals (CE, RM, LP), all with experience in autism research, identified through the research team’s professional networks joined the research team. As they participated in these research activities outside of their regular work duties, they were offered an honorarium. Members of the research team also included family members of autistic people (SP, RS, HW)  , and practitioners who currently engage in the assessment of autistic children, and/or currently or have previously provided professional supports to autistic children (SP, RS, HW). All members of the research team contributed to the following: (1) agreement on the research aim and design, (2) development of the survey materials, (3) dissemination of the survey, (4) discussion and decision making regarding the approach to data analysis, (5) interpretation of results, and (6) contributing to preparation of the manuscript.

## Results

### Demographics

The demographic information for 181 participants included in the analysis are provided in Table 1. Almost half the participants indicated their primary identity as parents (43.6%), followed by practitioners (29.3%), and autistic individuals (27.1%, of which 12% reported that they self-identified as autistic, or were currently undergoing diagnostic assessments for autism). Across the sample 35 participants (19.3%) identified across more than one participant group, see Table 1 for a breakdown of overlapping perspectives. Across all participants, the majority resided in Australia, were of European Australian heritage, and identified as female. The highest level of education attainment across the sample was a postgraduate degree. Practitioners worked across range of disciplines, including speech pathology, occupational therapy, psychology. For further detail see Table S3. No specific data on socioeconomic status was recorded for this sample. Note that Table 1 presents demographic information according to participants self-selected primary identity. Demographic characteristics unique to participant group are summarised in Tables S1-S3.

**Table 1:** Participant demographics across self selected identity groups

	All sample (*N* = 181)	Autistic (*n* = 49)	Parent (*n* = 79)	Practitioner (*n* = 53)
Participant identities				
Autistic only	43 (23.8)			
Autistic parent	16 (8.8)			
Autistic practitioner	7 (3.9)			
Autistic parent and	6 (3.3)			
practitioner				
Parent only	57 (31.5)			
Parent and practitioner	6 (3.3)			
Practitioner only	46 (25.4)			
Country				
Australia	154 (85.1)	41 (83.7)	67 (84.8)	46 (86.8)
New Zealand	27 (14.9)	8 (16.3)	12 (15.2)	7 (13.2)
Gender				
Female	150 (82.9)	33 (67.3)	66 (83.5)	51 (96.2)
Male	23 (12.7)	12 (24.5)	9 (11.5)	2 (3.8)
Non-binary	8 (4.4)	6 (12.2)	2 (2.5)	-
Other	1 (0.6)	1 (2.0)	-	-
Ethnic group				
New Zealand European	25 (13.8)	9 (18.4)	9 (11.4)	7 (13.2)
European Australian	133 (73.5)	37 (75.5)	58 (73.4)	38 (71.7)
Māori	3 (1.7)	-	2 (2.5)	1 (1.9)
Aboriginal	1 (0.6)	-	1 (1.3)	-
Chinese	5 (2.8)	1 (2.0)	-	4 (7.5)
Indian	3 (1.7)	1 (2.0)	1 (1.3)	1 (1.9)
Other	16 (8.84)	5 (10.2)	7 (8.9)	4 (7.5)
Prefer not to say	2 (1.1)	-	2 (2.5)	-
Education				
Primary/Intermediate	2 (1.1)	2 (4.1)	-	-
School				
College/High School	23 (12.7)	14 (28.6)	9 (11.4)	-
Trade/technical/vocational	25 (13.8)	5 (10.2)	19 (24.1)	1 (1.9)
training				
Bachelor’s/Undergraduate	57 (31.5)	16 (32.7)	21 (26.6)	20 (37.7)
University Degree				
Postgraduate University	69 (38.1)	10 (20.4)	27 (34.2)	32 (60.4)
Degree				
Other	2 (1.1)	1 (2.0)	1 (1.3)	-
Prefer not to say	1 (0.6)	1 (2.0)	-	-
Missing			2 (2.5)	

*Note* Demographic information presented for autistic individuals, parents, and practitioners correspond with their self-selected primary identity

### Perspectives on Early Supports

Table 2 outlines participant perspectives of early support provision. Across participants, the majority (59.2%) indicated ‘yes’ it was appropriate to provide early supports to autistic children, followed by participants (39.7%) who indicated ‘it depends’. Few participants (1.1%) indicated that it was not appropriate to provide early supports. Autistic individuals and practitioners were more likely to indicate that the provision of supports depended on the nature of those supports, compared to non-autistic individuals (χ2(1) = 13.98, *p* < .001, *V* = .281) and non-practitioners (χ^2^(1) = 4.08, *p* = .043, *V* = .152). A significantly greater proportion of parents indicated that early supports were appropriate, compared to non-parents (χ^2^(1) = 7.13, *p* = .008, *V* = .201).

**Table 2:** Participant perspectives on early supports

	All*	Autistic	Non-autistic		Parent	Non-parent		Practitioner	Non-practitioner	
	*n* (%)	*n* (%)	*n* (%)	*p*	*n* (%)	*n* (%)	*p*	*n* (%)	*n* (%)	*p*
Yes (appropriate)	106 (59.2)	30 (42.2)	76 (70.4)	< 0.001	59 (70.2)	47 (49.5)	0.008	32 (49.2)	74 (64.9)	0.043
No (not appropriate)**	2 (1.1)	1 (1.4)	1 (0.9)			2 (2.1)		1 (1.5)	1 (0.9)	
It depends	71 (39.7)	40 (56.3)	31 (28.7)		25 (29.8)	46 (48.4)		32 (49.2)	39 (34.2)	

*Missing *n* = 2

** Excluded from chi-square analysis

### Preferred Model of Disability

Table 3 outlines participant alignment with models of disability. Across groups, participants most often aligned with the strengths and challenges model (45.3%), followed by the biopsychosocial model (29.8%), and the social model (15.5%). Few participants reported alignment with the medical model (3.3%). For those who selected another model (5.5%), some commented that a combination of these models was the most appropriate and/or indicated a preference to support parents. While autistic individuals and parents were more likely to align with the strengths and challenges model, this did not significantly differ from non-autistic individuals and non-parents, respectively. Practitioners most frequently reported alignment with the biopsychosocial model, with a significantly smaller proportion reporting alignment with the strengths and challenges model of disability, compared to non-practitioners (χ^2^(3) = 18.23, *p* < .001, V = 0.318). See S5 for non-significant Chi-square test statistics.

**Table 3:** Participant perspectives on models of disability

	All*	Autistic	Non-autistic		Parent	Non-parent		Practitioner	Non-practitioner	
	*n* (%)	*n* (%)	*n* (%)	*p*	*n* (%)	*n* (%)	*p*	*n* (%)	*n* (%)	*p*
Medical model**	6 (3.3)	1 (1.4)	5 (4.6)	0.154	5 (5.9)	1 (1.0)	0.092	-	6 (5.2)	< 0.001
Social model	28 (15.5)	13 (18.1)	15 (13.8)		8 (9.4)	20 (20.8)		18 (27.7)	10 (8.6)	
Biopsychosocial model	54 (29.8)	15 (20.8)	39 (35.8)		29 (34.1)	25 (26.0)		24 (36.9)	30 (25.9)	
Strengths and challenges	82 (45.3)	38 (52.8)	44 (40.4)		37 (43.5)	45 (46.9)		20 (30.8)	62 (53.4)	
Another model**	10 (5.5)	5 (6.9)	5 (4.6)		5 (5.9)	5 (5.2)		3 (4.6)	7 (6.0)	

*Missing *n* = 1

**Combined responses for chi-square analysis into ‘other’ category

### Appropriateness of Outcomes

#### Child Outcomes

The results of McNemar’s tests highlighted no differences in whether child outcomes were considered appropriate or not according to child age. Participant appropriateness ratings were subsequently recoded into a single variable where a code of ‘not appropriate’ was assigned where participants had selected this response for children aged 0–5 years, and/or children aged 6–12 years. Table S6 presents the combined proportion of participants who indicated outcomes were *not* an appropriate target of professional supports for children.

All child outcomes were rated as not an appropriate target by at least one participant, except for *increasing access to sensory tools* and *improving the child’s understanding of themself*. Across all participant groups, the outcomes most frequently rated as not appropriate were: *reducing sensory seeking and avoidant behaviours* (53.6%), *reducing their focused interests* (54.0%), *social skills training* (48.9%), and *changing their play to appear more neurotypical* (49.6%). The following outcomes had sufficient variability to be included in Chi-Square analyses: *reducing sensory seeking and avoidant behaviours, reducing focused interests, social skills training, changing play skills*, and *increasing children’s knowledge of ways to be social.* Results of the analysis for these variables are presented in Table 4.

**Table 4:** Proportion of participants who indicated outcomes were not an appropriate outcome of support

	All	Autistic	Non-autistic	p	Parent	Non-parent	p	Practitioner	Non-practitioner	p
	n (%)	n (%)	n (%)		n (%)	n (%)		n (%)	n (%)	
Sensory behaviours	74 (53.6)	33 (58.9)	41 (50.0)	0.302	27 (40.9)	47 (65.3)	0.004	36 (73.5)	38 (42.7)	< 0.001
Focused interests	74 (54.0)	36 (63.2)	38 (47.5)	0.070	27 (41.5)	47 (65.3)	0.005	36 (73.5)	38 (43.2)	< 0.001
Social skills training	69 (48.9)	32 (57.1)	37 (43.5)	0.114	25 (36.8)	44 (60.3)	0.005	38 (74.5)	31 (34.4)	< 0.001
Play skills	67 (49.6)	31 (57.4)	36 (44.4)	0.140	20 (31.3)	47 (66.2)	< 0.001	37 (75.5)	30 (34.9)	< 0.001
Ways of being social	13 (9.2)	3 (5.3)	10 (11.8)	0.188	8 (11.6)	5 (6.8)	0.096	8 (15.7)	5 (5.5)	0.043

*Note* Only those outcomes included in the Chi-Square analysis are presented here. For a full breakdown of participant responses across all outcomes, see supplementary materials

A significantly greater proportion of parents (versus non-parents) indicated that the following outcomes were appropriate targets of supports: *reducing a child’s sensory seeking and avoidant behaviours (*χ^2^(1) = 8.22, *p* = .004, *V* = 0.244), *reducing a child’s focused interests* (χ^2^(1) = 7.75, *p* = .005, *V* = 0.238), *social skills training (*χ^2^(1) = 7.79, *p* = .005, *V* = 0.235), and *increasing a child’s use of neurotypical play skills* (χ^2^(1) = 16.44, *p* < .001, *V* = 0.349). Conversely, a significantly greater proportion of practitioners (versus non-practitioners) indicated that these outcomes were *not* appropriate targets of supports: *reducing a child’s sensory seeking and avoidant behaviours* χ^2^(1) = 12.03, *p* < .001, *V* = 0.295, *reducing a child’s focused interests* (χ^2^(1) = 11.62, *p* < .001, *V* = 0.291), *social skills training* (χ^2^(1) = 20.91, *p* < .001, *V* = 0.385), and *increasing a child’s use of neurotypical play skills* (χ^2^(1) = 20.61, *p* < .001, *V* = 0.391). There were no significant differences between groups for targeting the child’s *knowledge of different ways of being social*. Across all analyses, there were no differences between autistic and non-autistic participants. See S5 for non-significant Chi-square test statistics.

#### Parent Outcomes

All parent-focused outcomes were rated as appropriate outcomes of professional supports by all participants. No further analyses were conducted.

### Priority of Outcomes

#### Child Outcomes

Table 5 presents the mean priority score and ranking for child outcomes of the combined sample for autistic children aged 0–5 and 6–12 years. For mean priority scores according to participant group, see Tables S7 and S8. Across participants, the overall highest priority was unanimously *improving child mental wellbeing* (0–5 years: *M =* 3.73, *SD* = 0.70; 6–12 years: *M* = 3.84, SD = 0.54), and lowest priority was unanimously *reducing a child’s sensory seeking and avoidant behaviours* (0–5 years: *M =* 1.38, *SD* = 0.76; 6–12 years: *M* = 1.62, SD = 0.99). Results of the Wilcoxon signed-rank tests revealed significant (at *p* ≤ .01) median differences in priority ratings for six of the 20 child outcomes common across age groups (see S5). There were statistically significant median increases in priority ratings for outcomes of professional supports for children aged 6–12 years including: *improvements in a child’s understanding of themselves (*z = 3.78, *p* < .001), *reducing a child’s sensory seeking and avoidant behaviours (*z = 2.81, *p* = .005), *improvements in a child’s social-emotional skills* (z = 5.40, *p* < .001), *improvements in a child’s academic/school skills* (z = 3.03, *p* = .002), *improvements in a child’s self-care skills* (z = 3.89, *p* < .001), and the *child’s satisfaction with supports* (z = 2.58, *p* = .010).

**Table 5:** Mean priority ratings of outcomes for autistic children aged 0–5 and 6–12 years

	Children aged 0–5 years			Children aged 6–12 years		
Outcomes	*n*	*Mean (SD)*	Rank	*n*	*Mean (SD)*	Rank
Mental wellbeing	153	3.73 (0.70)	1	139	3.84 (0.54)	1
Behaviours that harm self and/or others	149	3.54 (0.93)	2	133	3.68 (0.81)	4
Physical wellbeing	153	3.52 (0.90)	3	138	3.59 (0.83)	5
Self-advocacy skills	-	-	-	142	3.70 (0.73)	3
Inclusivity and accessibility	170	3.51 (0.89)	4	144	3.58 (0.84)	6
Understanding self	164	3.43 (0.93)	5	142	3.72 (0.70)	2
Sensory tools	169	3.38 (0.96)	6	142	3.51 (0.89)	7
Satisfaction with supports	149	3.34 (1.00)	7	139	3.50 (0.94)	8
Expressive language	161	3.27 (1.03)	8	140	3.25 (1.03)	11
Receptive language	162	3.04 (1.07)	9	141	2.99 (1.05)	12
Self-care skills	150	3.01 (1.06)	10	136	3.27 (1.00)	10
Social emotional skills	152	3.01 (1.07)	11	136	3.49 (0.89)	9
Cognition	161	2.87 (1.12)	12	142	2.93 (1.04)	14
Participation	152	2.84 (1.07)	13	135	2.96 (1.08)	13
Motor skills	144	2.68 (1.04)	14	136	2.70 (1.04)	16
Knowing ways of being social	163	2.44 (1.15)	15	143	2.72 (1.10)	15
School skills	153	2.16 (0.96)	16	136	2.50 (1.03)	17
Social skills training	162	1.77 (1.09)	17	141	1.95 (1.19)	18
Play	152	1.76 (1.05)	18	136	1.84 (1.10)	19
Focused interests	159	1.53 (0.86)	19	139	1.69 (0.98)	20
Sensory behaviours	160	1.38 (0.76)	20	139	1.62 (0.99)	21

Table S9 presents the results of a series of standard multiple regressions examining participant identity, perspectives on early supports, and models of disability as predictors of child outcomes. For outcomes where no differences existed in priority ratings for children aged 0–5 and 6–12 years, priority scores were averaged for analysis. Further, the following outcomes were not included in analysis due to limited variation in responses: *understanding self* (6–12 years), *improvements in social emotional skills* (6–12 years), *satisfaction with supports* (6–12 years), *behaviours that harm self and/or others* (average), *improvements in mental wellbeing* (average), and *improvements in self-advocacy skills* (6–12 years). The combined variables were significant predictors of mean priority scores for the following outcomes: *inclusivity and accessibility, F*(7, 158) = 5.60, *p* < .001, R^2^ = 0.199; *sensory tools, F*(7, 157) = 3.39, *p* = .002, R^2^ = 0.131; *improvements in cognition, F*(7, 152) = 5.40, *p* < .001, R^2^ = 0.199, *improvements in receptive language, F*(7, 153) = 3.45, *p* = .002, R^2^ = 0.136; *social skills training, F*(7, 150) = 11.77, *p* < .001, R^2^ = 0.354; *knowledge of different ways of being social*, F(7, 152) = 3.67, *p* < .001, R2 = 0.145; *reducing a child’s focused interests*, F(7, 149) = 8.03, *p* < .001, R2 = 0.274, *improvements in motor skills*, F(7, 139) = 3.82, *p* < .001, R2 = 0.161, *participation*, F(7, 141) = 3.61, *p* < .001, R2 = 0.152; *increasing a child’s use of neurotypical play skills*, F(7, 142) = 7.04, *p* < .001, R2 = 0.258, *physical wellbeing*, F(7, 142) = 4.12, *p* < .001, R2 = 0.169; *reducing a child’s sensory seeking and avoidant behaviours (0–5 years)*, F(8, 141) = 6.52, *p* < .001, R2 = 0.270, *reducing a child’s sensory seeking and avoidant behaviours (6–12 years)*, F(7, 129) = 7.28, *p* < .001, R2 = 0.283, *child’s satisfaction with supports (0–5 years)*, F(7, 139) = 5.83, *p* < .001, R2 = 0.227. The R^2^ values ranged from 0.136 for *improvements in receptive language*, and 0.354 for *social skills training*, indicating that the models explained between 13.6 and 35.4% of the variance in mean priority scores.

Participant identity was a significant predictor of priority ratings for several child outcomes. Mean priority scores for *improving inclusivity and accessibility* were higher for parents (versus non-parents). Mean priority scores for *reducing sensory seeking and avoidant behaviours* (0–5 years), *reducing focused interests*, *improving cognitive skills*, and *social skills training* were significantly lower for practitioners (versus non-practitioners), with higher mean priority scores for *improving inclusivity and accessibility*, and *improving access to sensory tools*. Participant perspectives on whether it was appropriate to provide early supports to autistic children also predicted priority scores for some outcomes. Mean priority scores for *social skills training, improving child’s understanding of ways to be social*, *reducing focused interests*, and *reducing sensory seeking and avoidant behaviours* (0–5 and 6–12 years), *changing the child’s play to reflect neurotypical norms*, and *improvements in the child’s participation* were higher for participants who considered providing early supports as appropriate, compared to participants who reported that it depended on the type of support. Mean priority scores for the *child’s satisfaction with supports* (0–5 years) were lower for participants who considered providing early supports as appropriate, compared to participants who reported that it depends. Participants’ preferred model of disability was also a significant predictor of outcomes. Mean priority scores for *improving inclusivity and accessibility* and the *child’s satisfaction with supports* (0–5 years) were lower for participants who aligned with the biopsychosocial model of disability, compared to those who aligned with the social model. Mean priority scores for *improving physical wellbeing* were higher for participants who aligned with the strengths and challenges model, compared to those who aligned with the social model.

#### Parent Outcomes

Table 6 presents the mean priority score and ranking for parent outcomes (see Table S10 for ratings across participant groups). While all parent outcomes were rated between a medium to high priority, across participants the overall highest priority was unanimously *improving parent knowledge of autism* (*M* = 3.78, *SD* = 0.62), and lowest priority was unanimously *improving parent physical wellbeing* (*M* = 3.26, *SD* = 1.01). Table S11 presents the results of a series of standard multiple regressions examining participant identity and perspectives on early supports and models of disability as predictors of priority of parent outcomes. Parent *mental wellbeing*, *knowledge of autism*, and *responsiveness* were excluded from analyses due to limited variation. The combined variables were a significant predictor of *improving parent competence* (*F*(7, 128) = 2.28, *p* = .010, R^2^ = 0.132) and *satisfaction with supports* (*F*(7, 127) = 2.33, *p* = .010, R^2^ = 0.133), with R^2^ values of 0.132 and 0.133 indicating that the models explained approximately 13% of the variance in these parent outcomes. Mean priority scores for *parent satisfaction with supports* were higher for practitioners, compared to non-practitioners.

**Table 6:** Mean Priority Ratings of Outcomes for Parents of Autistic Children

	All	
	*n*	*Mean (SD)*
Knowledge of autism	139	3.78 (0.62)
Parent responsiveness	138	3.61 (0.82)
Parent mental wellbeing	136	3.55 (0.85)
Parent sense of competence	138	3.41 (0.95)
Parent satisfaction with supports	137	3.41 (0.95)
Parent physical wellbeing	135	3.26 (1.01)

## Discussion

It is essential to seek stakeholder’s perspectives on supports for autistic children. This was the first study to examine the perceptions of the autistic and autism community regarding the appropriateness and priority of targeted outcomes in the provision of professional supports for autistic children. In the current study, the most appropriate and highest priority outcomes across participant groups largely related to the child’s wellbeing and quality of life. Further, outcomes which were most frequently rated as not appropriate, and of the lowest priority, were those which seek to change characteristic linked with a child’s autistic identity, often in ways that make them appear more neurotypical.

This is largely consistent with the findings of Waddington et al. (2024). However, practitioners, rather than autistic people, in the current sample were most likely to perceive outcomes which target autism characteristics as both inappropriate and a low priority. Further, both practitioners and autistic individuals were more likely to suggest that the appropriateness of providing early supports to autistic children depended on the nature of those supports, where this was true only for autistic participants in Waddington et al. (2024). These findings support increasing neurodiversity-affirming practice, particularly among practitioners and within a short two-year period since Waddington et al. (2024) conducted their research. This might be explained in part by the growing demand for practitioners to provide strengths-based approaches to professional supports (Dawson et al., 2022). Further, in the Australian and New Zealand context of this study, a number of factors might have influenced these findings, including the focus on neurodiversity-affirming practice in recently released practitioner focused guidelines (Trembath et al., 2022; Whaikaha and Ministry of Education, 2022), and professional body conferences (Australian Psychological Society, 2023).

The alignment with more neurodiversity-affirming perspectives on providing professional supports and targeted outcomes was less obvious among parents surveyed. While many parents agreed that targeting outcomes which seek to change aspects of the child that are linked with autistic identity were not appropriate (i.e., *reducing a child’s sensory seeking and avoidant behaviours, reducing a child’s focused interests, social skills training*, and *increasing a child’s use of neurotypical play skills*), they were more likely than non-parents in the sample to rate these as appropriate outcomes of support. This may reflect the real world challenges parents experience when trying to support the learning, participation, and wellbeing of their child, with the early years further emphasised as a ‘critical’ period of development (Edwards et al., 2017). Indeed, recent research suggests that parents strongly desire access to professional supports for their child during early childhood as they see this as the greatest opportunity improving future outcomes (Bent et al., 2023). Further, some tensions have been suggested to exist between parents and autistic advocates, particularly in relation to the types of targeted outcomes for autistic children with co-occurring diagnoses who might relatedly have more complex support needs (Leadbitter et al., 2021). While many parents reported their child having a co-occurring diagnosis (64.8%) in the current study, information was not collected about the specific support needs of the children being reported on, so it is unclear if this sentiment is reflected in the current sample. Further, it is likely that a diverse range of views regarding outcomes are experiences by parents of children both with and without co-occurring diagnoses.

To our knowledge, this is the first study to explore whether perspectives regarding the appropriateness and importance of child outcomes change over time. While appropriateness of the selected outcomes did not change across child age ranges in the current sample, significantly higher mean priority ratings were observed for six of the 20 outcomes for children aged 6–12 years versus 0–5 years (i.e., *improvements in a child’s understanding of themselves, reducing a child’s sensory seeking and avoidant behaviours*, *improvements in a child’s social-emotional skills*, *improvements in a child’s academic/school skills*, *improvements in a child’s self-care skills*, and the *child’s satisfaction with supports)*. These findings are consistent with the increased developmental and academic expectations that are likely to be placed on children as they move from early childhood into primary school settings. Primary classroom settings are generally more structured than early childhood settings and children are often required to participate in standardised assessment, which may contribute to greater expectations of independence and conformity with peers (Larcombe et al., 2019; New Zealand Council for Educational Research, 2022).

A novel element of this study was the inclusion of outcomes related to parents. Parent support outcomes are rarely reported (see Trembath et al., 2023), despite research demonstrating the increased incidence of mental health difficulties including depression and parenting-related stress (Ingersoll & Hambrick, 2011; Quintero & McIntyre, 2010) among parents of autistic children. The present study found that all parents’ outcomes (i.e., *physical and mental wellbeing, knowledge of autism, competence, responsiveness to their child*, and *overall satisfaction with supports*) were unanimously considered appropriate targets and of a medium to high priority, emphasising the need for outcomes to extend beyond the child to consider the broader family unit (Estes et al., 2019).

There are several opportunities for subsequent work to address the limitations of the present study. While this survey included parents of autistic children, it is difficult to draw conclusions about the types of outcomes that are most important to the children themselves who are receiving supports. Further research should utilise developmentally appropriate methodologies to seek the perspectives of autistic children themselves. While a reasonable proportion of autistic individuals were included in the current study, those who participated had largely not accessed professional supports during childhood. This may be due to a combination of the average age of diagnosis being 28 years in the current sample, or a lack of available supports during their childhood. Further, while the current sample reported a range of co-occurring diagnoses (e.g., intellectual disability, anxiety), future research may seek to directly recruit a diverse range of autistic individuals to determine whether these findings reflect the views of the broader autistic population. Additionally, we included those participants who self-identified as being autistic or were currently undergoing assessment for a diagnosis of autism, in our autistic cohort of participants. While this was a small proportion of all autistic participants (12%), it is possible that their perspectives may differ in a meaningful way from individuals who have already received a formal diagnosis of autism. Future research may choose to investigate these subgroup differences. Unlike Waddington et al. (2024), Māori, Aboriginal, and Torres Strait Islander peoples were not well represented in the current study so conclusions cannot be drawn about outcome priorities for these indigenous populations. Research which includes the application of culturally-appropriate approaches (e.g., Kaupapa Māori research) to examine appropriateness and priorities of early support outcomes for Indigenous and other culturally diverse populations is therefore an important future avenue. It is also possible that the choice to describe some outcomes as a reduction in a specific area (i.e., *reducing a child’s sensory seeking and avoidant behaviours*) may have biased participants towards a particular response when indicating its appropriateness and priority level. Finally, given the self-report nature of the data collected, it is possible that participants’ general views may not reflect what is actively prioritised in their daily lives or professional practice. As our findings indicate that some outcomes become a higher priority as children get older, future research might seek to understand these changes and whether this is consistent as children move into later developmental stages, such as adolescence. Finally, these findings add to the body of literature supporting both research and practice that encourages prioritisation of those outcomes which are meaningful to the autistic and autism communities.

## Electronic supplementary material

Below is the link to the electronic supplementary material.

Supplementary Material 1

## Declarations

The authors would like to thank members of the autistic and autism communities who took the time to share their perspectives.
