# Supplementary material for: Community Priorities for Outcomes Targeted During Professional Supports for Autistic Children and their Families
Source: J Autism Dev Disord. 2024 Apr 20;55(5):1890–901. doi: 10.1007/s10803-024-06333-2 (PMC12021951; doi:10.1007/s10803-024-06333-2)
Supplement: Supplementary file 1 — Supplementary Material 1 [file 10803_2024_6333_MOESM1_ESM.docx]

**Supplementary Materials**

**Table S1.**

*Demographic Information of Autistic Adults*

| Characteristic | *n* (%) |
| --- | --- |
| Age (years)  16-25  26-30  31-40  41-50  Over 50 | 13 (26.5)  10 (20.4)  10 (20.4)  5 (10.2)  11 (22.4) |
| Diagnostic status  Formal diagnosis of autism  Currently being assessed  Self-identify | 43 (87.8)  1 (2.0)  5 (10.2) |
| Age of formal diagnosis | 28.42 years (16.16 years)  Range 3 – 71 years |
| Other diagnoses  Yes  No  Prefer not to say  Co-occurring diagnoses  Anxiety  Ataxia  ADHD/ADD  Depression  GDD Intellectual disability  OCD  Other | 37 (75.5)  11 (22.4)  1 (2.0)  27 (55.1)  1 (2.0)  15 (30.6)  23 (46.9)  2 (4.1)  2 (4.1)  7 (14.3)  15 (30.6) |
| Supports during 0-5 years  Yes  Yes, unsure of the specific support  No  Don’t know  Includes:  Speech therapy  Occupational therapy  Psychologist  Music therapy | 2 (4.1)  3 (6.1)  42 (85.7)  2 (4.1)  1 (2.0)  1 (2.0)  1 (2.0)  1 (2.0) |
| Supports during 6-12 years  Yes  Yes, unsure of the specific support  No  Includes:  Speech therapy  Occupational therapy  Social skills supports | 3 (6.1)  4 (8.2)  42 (85.7)  3 (6.1)  1 (2.0)  2 (4.1) |

Abbreviations: ADHD/ADD - attention deficit hyperactivity disorder/attention deficit disorder, GDD – global developmental delay, OCD – obsessive compulsive disorder

**Table S2.**

*Demographic Information Provided by Parents Of Autistic Children*

| Characteristics | n (%) |
| --- | --- |
| Relationship to child  Mother  Father | 82 (90.1)  9 (9.9) |
| Age of child  0-5 years  6-12 years  13-18 years  19+ years | 14 (15.4)  48 (52.7)  23 (25.3)  6 (6.6) |
| Child’s gender  Female  Male  Non-binary  Other | 28 (30.8)  60 (65.9)  1.0 (1.1)  2.0 (2.2) |
| Formal diagnosis  Yes  No | 89 (97.8)  2 (2.2) |
| Other diagnoses  Yes  No  I prefer not to say | 59 (64.8)  30 (33.0)  1.0 (1.1) |
| Co-occurring diagnoses  Anxiety  Ataxia  ADHD/ADD  Depression  GDD  Intellectual Disability  OCD  Other | 40 (44.0)   1. (1.1)   32 (35.2)  9 (9.9)  9 (9.9)  7 (7.7)  6 (6.6)  19 (20.9) |
| Child’s ethnic group  New Zealand European  European Australian  Māori  Aboriginal  Torres Strait Islander  Niuen  Indian  Other  Prefer not to say | 11 (12.8)  72 (79.1)  6 (6.6)  3 (3.3)  1.0 (1.1)  1.0 (1.1)  1.0 (1.1)  4 (4.4)  2 (2.2) |
| Supports provided 0-5 years  Yes  No, does not currently receive supports  No, did not receive supports between 0-5 years  Prefer not to say | 52 (57.1)  2 (2.2)  36 (39.6)  1 (1.1) |
| Types of early supports  Speech therapy  Occupational therapy ABA  Psychology  Play therapy  Art therapy  Music therapy  Physiotherapy  Relationship based therapy  Social skills programs/therapy  Cognitive behavioural therapy  Parent education programs  Behaviour support  Multidisciplinary therapies  Other | 44 (48.4%)  40 (44.0)  10 (11.0)  18 (19.8)  10 (11.0)  3 (3.3)  4 (4.4)  12 (13.2)  2 (2.2)  10 (11.0)  2 (2.2)  25 (27.5)  8 (8.8)  6 (6.6)  6 (6.6) |
| Supports provided 6-12 years  Yes  Child currently 0-5 years, supports listed previously  No, did not receive supports between 6-12 years  No, does not currently receive supports | 54 (59.3)  15 (16.5)  14 (15.4)  9 (9.9) |
| Types of early supports  Speech therapy  Occupational therapy ABA  Psychology  Play therapy  Art therapy  Music therapy  Physiotherapy  Social skills programs/therapy  Cognitive behavioural therapy  Parent education programs  Behaviour support  Multidisciplinary therapies  Other | 32 (35.2)  41 (45.1)  2 (2.2)  34 (37.4)  5 (5.5)  6 (6.6)  8 (8.8)  4 (4.4)  19 (20.9)  10 (11.0)  16 (17.6)  13 (14.3)  3 (3.3)  4 (4.4) |

Note: Information provided for 91 children

Abbreviations: ADHD/ADD - attention deficit hyperactivity disorder/attention deficit disorder, GDD – global developmental delay, OCD – obsessive compulsive disorder

**Table S3.**

*Demographic Information of Practitioners*

| Characteristics | *n* (%) |
| --- | --- |
| Role  Psychologist  Occupational therapist  Speech pathologist  Social worker  Behaviour therapist (not board certified)  Researcher  Other | 10 (18.9)  9 (17.0)  19 (35.8)  2 (3.8)  4 (7.5)  4 (7.5)  5 (9.4) |
| Time in current role | 7.39 years (7.31 years)  Range 1 – 37 years |
| Time spent working with autistic children | 9.80 years (8.36 years)  Range 1 – 35 years |
| Age groups worked with  0-5 years  6-12 years  13-18 years  19+ years | 42 (79.2)  47 (88.7)  35 (66.0)  24 (45.3) |
| Settings  Hospital  Community clinic (inc. private practice)  University clinic  Other | 2 (3.8)  40 (75.5)  3 (5.7)  19 (35.8) |

**S4.** Copy of survey

**Section 1. Eligibility Criteria**

- 1. Which country do you currently live in?
- Australia
- New Zealand
- Other [BRANCHING LOGIC – DISCONTINUE]
  1. Which of the following describes you? [select all that apply]
- I am an autistic person aged 16 years and over.
- I am a parent or caregiver of an autistic child under 18 years.
- I am a practitioner (including researchers) working with autistic children under 18 years.
- None of the above [BRANCHNG LOGIC – DISCONTINUE]

[If multiple selections made above]

- 1. Please choose the main point of view you want to use when answering the questions in this survey.

*Please note that this is primarily for the purpose of analysis, and to help reduce the time spent responding to demographic information.*

- I am an autistic person aged 16 years and over.
- I am a parent or caregiver of an autistic child under 18 years.
- I am a practitioner (including researchers) working with autistic children under 18 years.

**Discontinue message:**

Thank you very much for your interest in this study. At this time, we are specifically looking for autistic people aged 16 years and over, parents of autistic children aged 18 years and under, and professionals working with children aged 18 years and under living in New Zealand or Australia. Unfortunately, the answers you provided suggest that you do not meet our current criteria for participation. We appreciate your interest in this research and thank you for your time.

**Section 2: Demographics**

2.1 With which gender do you identify? (check all that apply)

- Female
- Male
- Non-binary
- Other, please specify
- Prefer not to say

2.2 Which ethnic group(s) do you belong to? Please select all which apply to you

- New Zealand European
- European Australian
- Māori
- Aboriginal
- Torres Strait Islander
- Samoan
- Cook Islands Māori
- Tongan
- Niuean
- Chinese
- Indian
- Vietnamese
- Philippine
- Other e.g., Japanese, Tokelauan (Please state):___________________
- Prefer not to say

2.3 What is the highest level of education you have completed?

- Primary/Intermediate School
- College/High School
- Trade/technical/vocational training
- Bachelor’s/Undergraduate University Degree
- Postgraduate University Degree
- Other (Please describe): _______________
- Prefer not to say

**Autistic Individuals**

3.1 How old are you?

- 16-25
- 26-30
- 31-40
- 41-50
- Over 50
- Prefer not to say

3.2 Which of the following best describes your circumstances?

- I have a formal diagnosis of autism (or a related diagnosis e.g., Asperger’s, Pervasive-Developmental Disorder) made by a qualified health practitioners (e.g., paediatrician, clinical psychologist, psychiatrist) [BRANCH LOGIC]
  - At what age did you receive a formal diagnosis?

*(free text response)*

- I am currently being assessed for a possible autism diagnosis
- I self-identify as autistic, but have not been given and/or am not seeking a formal diagnosis by a health practitioner.
- Prefer not to say

3.3 Do you have any other clinical diagnoses (this can include both psychiatric and physical conditions) in addition to autism? Please select all which apply to you.

- Anxiety
- Ataxia
- Attention deficit hyperactivity disorder or attention deficit disorder
- Depression
- Global developmental delay
- Intellectual disability
- Obsessive Compulsive Disorder
- Other; Please state:___________
- No other diagnoses
- I prefer not to say

3.4 Did you receive any professional autism supports during early childhood (aged between 0-5 years)*?

**These supports might also be called interventions or therapy. For this study, these supports do not include respite care or support by your child’s classroom teacher/educator.*

- Yes (please select all that apply):
  - Speech and language therapy
  - Occupational therapy
  - ABA-based therapy
  - Psychologist support
  - Drama therapy
  - Play therapy
  - Art therapy
  - Music therapy
  - Physiotherapy
  - Relationship-based therapy
  - Social skills support programs or therapies
  - Cognitive behavioural therapy (CBT)
  - Parent education/training programmes
  - Behaviour support
  - Multidisciplinary interventions
  - Other (please state):
- Yes, but I am unsure of the specific supports
- No
- I was not yet diagnosed
- I don’t know
- Prefer not to say

3.4a How many hours of support did you receive on average across all services per month?

_________________ hours

3.5 Did you receive any professional autism supports during primary school years (aged between 6-12)?

- Yes (please select all that apply):
  - Speech and language therapy
  - Occupational therapy
  - ABA-based therapy
  - Psychologist support
  - Drama therapy
  - Play therapy
  - Art therapy
  - Music therapy
  - Physiotherapy
  - Relationship-based therapy
  - Social skills support
  - Cognitive behavioural therapy (CBT)
  - Parent education/training programmes
  - Behaviour support
  - Multidisciplinary interventions
  - Other (please state):
- Yes, but I am unsure of the specific supports
- No
- I was not yet diagnosed
- I don’t know
- Prefer not to say

3.5a How many hours of support did you receive on average across all services per month?

_________________ hours

**Parent/Caregiver**

If you are a parent of more than one autistic child, please respond to the following questions for one child at a time. You will be given the option to add details for additional children below.

4.1 What is your relationship to the child?

- Mother
- Father
- Other, please specify (e.g., birthing parent, grandmother, foster carer)

4.2 How old is your child?

- 0-5 years
- 6-12 years
- 12-18 years

4.3What is your child’s gender?

- Female
- Male
- Non-binary
- Other, please specify
- Prefer not to say

4.4 Has your child received a formal diagnosis of autism (or a related condition e.g., Asperger’s, Pervasive Developmental Disorder)?

- Yes [BRANCHING LOGIC]

At what age was your child diagnosed? __________

- No
- Prefer not to say

4.5 Does your child have any of the following clinical diagnoses (this can include both psychiatric and physical conditions) in addition to autism? Please select all which apply.

- Anxiety
- Ataxia
- Attention deficit hyperactivity disorder or attention deficit disorder
- Depression
- Global developmental delay
- Intellectual disability
- Obsessive Compulsive Disorder
- Other; Please state:___________
- No other diagnoses
- I prefer not to say

4.6 Which ethnic group(s) does your child belong to? Please select all which apply.

- New Zealand European
- European Australian
- Māori
- Aboriginal
- Torres Strait Islander
- Samoan
- Cook Islands Māori
- Tongan
- Niuean
- Chinese
- Indian
- Vietnamese
- Philippine
- Other e.g., Japanese, Tokelauan (Please state):___________________
- Prefer not to say

4.7 Did your child receive professional autism supports between the ages of 0-5 years*?

**These supports might also be called interventions or therapy. For this study, these supports do not include respite care or support by your child’s classroom teacher/educator.*

- Yes (please select all that apply):
  - Speech and language therapy
  - Occupational therapy
  - ABA-based therapy
  - Psychologist support
  - Drama therapy
  - Play therapy
  - Art therapy
  - Music therapy
  - Physiotherapy
  - Relationship-based therapy
  - Social skills support
  - Cognitive behavioural therapy (CBT)
  - Parent education/training programmes
  - Behaviour support
  - Multidisciplinary interventions
  - Other (please state):
- No, my child does not currently receive professional supports
- Prefer not to say

4.7a How many hours of support does your child receive on average across all services per month?

_________________ hours

4.8 Did your child receive professional autism supports between the ages of 6-12 years?

- Yes (please select all that apply):
  - Speech and language therapy
  - Occupational therapy
  - ABA-based therapy
  - Psychologist support
  - Drama therapy
  - Play therapy
  - Art therapy
  - Music therapy
  - Physiotherapy
  - Relationship-based therapy
  - Social skills support
  - Cognitive behavioural therapy (CBT)
  - Parent education/training programmes
  - Behaviour support
  - Multidisciplinary interventions
  - Other (please state):
- My child is currently aged 0-5 years, so their professional supports are listed above.
- No, my child did not receive professional support when they were aged 6-12 years/My child is currently 6-12 years and has not received any supports
- Prefer not to say

4.8a [If participants answered yes] How many hours of professional support services did your child receive across all services per month?

_________________ hours

**[Repeat questions if more than one child x 4]**

**Practitioner**

5.1 What is your current role?

- General practitioner
- Nurse
- Paediatrician
- Psychiatrist
- Psychologist
- Occupational therapist
- Speech Pathologist
- Drama therapist
- Play therapist
- Music therapist
- Art therapist
- Social Worker
- Physiotherapist
- Board certified behaviour analyst
- Behaviour therapist (not board certified)
- Researcher
- Other

5.2 If you are dual qualified, or work across more than one position, please indicate any additional roles here (select all that apply):

- General practitioner
- Nurse
- Paediatrician
- Psychiatrist
- Psychologist
- Occupational therapist
- Speech Pathologist
- Drama therapist
- Play therapist
- Music therapist
- Art therapist
- Social Worker
- Physiotherapist
- Board certified behaviour analyst
- Behaviour therapist (not board certified)
- Researcher
- Other

5.3 How long have you been in your current role?

________________years

5.4 How many years of experience do you have working in clinical practice with autistic children?

________________years

5.5 What age groups do you currently provide autism specific supports to (select all that apply):

- 0-5 years
- 6-12 years
- 12-18 years
- 19+ years

5.6 In which of the following settings do you currently provide supports to autistic children? (select all that apply)

- Hospital (inpatient/outpatient)
- Community clinic (including private practice)
- University clinic (includes providing services as part of student training and research)
- Other (please specify) __________________

**Section 3: Perspectives of early professional supports**

**1.** Do you personally believe that it is okay to provide early supports* to autistic children (aged between 0-12 years)?

** These supports might also be called interventions or therapy and may include things like speech and language therapy, occupational therapy, ABA-based therapy, psychologist support, etc.*

- Yes
- No
- Only some supports/it depends on the purpose of those supports
- Don’t know
- Prefer not to say

Do you wish to tell us more about this? (optional free text response)

_____________________

**2.** Which of these best aligns with your personal views of what early supports should focus on?

Early supports should focus on:

- Changing things about the child to reduce their disability and delays.
- Making specific changes to environments around the child, along with broader changes to society.
- Changing aspects about both the child’s development and their environment, and how these work together to support the child.
- Helping the child identify and understand their own strengths and difficulties, to self-discover helpful strategies and advocate for themselves.
- None of these align with my views, and I think early supports should focus on: ________________ (optional free text response)

**Section 4: Outcome Prioritisation**

In this section we will ask about the broad outcomes that we hope might improve as the result of a child receiving supports. We are not asking about the ways in which these improvements might be *measured* (i.e., increased scores on a measure of vocabulary).

We will be asking you if you think these outcome areas are appropriate and/or how importance they are to target for autistic children in general (not for yourself or your child if you have indicated that you are autistic yourself, or a parent of an autistic child).

We understand that you are answering with your own opinion, rather than on behalf of all autistic adults/parents/professionals. Please note that some of the terminology used below has been taken from diagnostic criteria and may not be the preferred language of the autistic community.

Note that we will ask you:

- Which outcomes are appropriate and a priority for 0-5 year olds receiving supports,
- Which outcomes are appropriate and a priority for 6-12 year olds receiving supports (this list is very similar to those for 0-5 year olds), and
- Which outcomes are appropriate and a priority for parents of autistic children receiving supports.

**Q1. Outcomes that may be targeted for autistic children aged between 0-5 years receiving supports.**

|  | Not an appropriate outcome of support | An appropriate outcome of support, and a | | | | No opinion/not sure |
| --- | --- | --- | --- | --- | --- | --- |
|  |  | Not at all a priority | Low priority | Medium priority | High Priority |  |
| **Inclusivity and accessibility of environments**:  Changing the physical and/or social environment to improve the  inclusion and wellbeing of the child (e.g., having sensory friendly public spaces). |  |  |  |  |  |  |
| **Sensory tools:**  Improving access to sensory tools that improve inclusion and wellbeing (e.g., ear defenders, fidget toys). |  |  |  |  |  |  |
| **Understanding and using**  **Information (also known as cognition):**  Improvements in how the child processes information (e.g., their attention, memory, decision making skills) |  |  |  |  |  |  |
| **Understanding language (also known as receptive language):**  Improvements in the child's understanding of other people's communication (e.g., increases in understanding instructions,  words, and stories). |  |  |  |  |  |  |
| **Using language (also known as expressive language):**  Improvements in the child's use of language (e.g., increased use of spoken language or gestures, or augmented and alternative  communication modes to express themselves). |  |  |  |  |  |  |
| If you wish to provide more information about your responses, please enter here. |  |  |  |  |  |  |
| **Understanding self:**  Increases in the child's ability to understand their wants and needs (e.g., learning what makes them happy or sad). |  |  |  |  |  |  |
| **Social skills training:**  Changes in the child's use of certain social skills (e.g., increasing eye contact, initiating conversations in a certain way). |  |  |  |  |  |  |
| **Knowing ways of being social:**  Increasing the child's knowledge of different ways of being social (e.g., some people want eye-contact while other people prefer not to use eye-contact) without the expectation for the child to be social in a certain way. |  |  |  |  |  |  |
| **Focused Interests:**  Decreases in the child's repetitive and focused interests (e.g., increasing range of interests, increasing flexibility in routines). |  |  |  |  |  |  |
| **Sensory behaviours:**  Decreases in the child's sensory seeking (e.g., fidgeting, moving body  frequently) and avoiding (e.g., covering ears, avoiding touch) behaviours. |  |  |  |  |  |  |
| If you wish to provide more information about your responses, please enter here. |  |  |  |  |  |  |
| **Social-emotional skills**:  Improvements in the child’s understanding, expression, and regulation of their emotions. |  |  |  |  |  |  |
| **Body movements (also known as motor skills)**:  Improvements in the child's body movements (e.g., use of hands, ability to use objects, walking, running, balance) |  |  |  |  |  |  |
| **Early school skills:**  Improvements in the child's skills in areas that reflect the education curriculum (e.g., number and letter recognition). |  |  |  |  |  |  |
| **Participation**:  Improvements in skills which help the child take part in daily activities (e.g., increasing their ability to transition between  environments, join in with group activities, and attend events within their community). |  |  |  |  |  |  |
| **Self-care skills:**  Improvements in skills that help the child look after themselves in their everyday life (e.g., feeding, getting dressed, toileting). |  |  |  |  |  |  |
| **Play skills:**  Increases in the child's use of neurotypical play skills including using toys for their intended purpose, playing alongside others, and playing pretend. |  |  |  |  |  |  |
| If you wish to provide more information about your responses, please enter here. |  |  |  |  |  |  |
| **Behaviours that harm self and/or others**:  Decreases in the child's behaviours that harm themselves and others (e.g., hitting, head-banging). |  |  |  |  |  |  |
| **Physical wellbeing:**  Improvements in the child’s physical wellbeing (e.g., their overall health, physical safety). |  |  |  |  |  |  |
| **Mental wellbeing:**  Improvement in the child’s mental wellbeing (e.g., happiness, control over one's life, confidence, time to engage in special interests). |  |  |  |  |  |  |
| **Satisfaction with support:**  What the child thinks about whether the support they are receiving is good or not. |  |  |  |  |  |  |
| If you wish to provide more information about your responses, please enter here. |  |  |  |  |  |  |

**Q2. Outcomes for children aged between 6-12 years receiving supports.**

|  | Not an appropriate outcome of support | An appropriate outcome of support, and a | | | | No opinion/not sure |
| --- | --- | --- | --- | --- | --- | --- |
|  |  | Not at all a priority | Low priority | Medium priority | High Priority |  |
| **Inclusivity and accessibility of environments**:  Changing the physical and/or social environment to improve the  inclusion and wellbeing of the child (e.g., having sensory friendly public spaces). |  |  |  |  |  |  |
| **Sensory tools:**  Improving access to sensory tools that improve inclusion and wellbeing (e.g., ear defenders, fidget toys). |  |  |  |  |  |  |
| **Understanding and using Information (also known as cognition):** Improvements in how the child processes information (e.g., their attention, memory, decision making skills) |  |  |  |  |  |  |
| **Understanding language (also known as receptive language):**  Improvements in the child's understanding of other people's  communication (e.g., increases in understanding instructions,  words, and stories). |  |  |  |  |  |  |
| **Using language (also known as expressive language):**  Improvements in the child's use of language (e.g., increased use of spoken language or gestures, or augmented and alternative  communication modes to express themselves). |  |  |  |  |  |  |
| If you wish to provide more information about your responses, please enter here. |  |  |  |  |  |  |
| **Understanding self:**  Increases in the child's ability to understand their wants and needs (e.g., learning what makes them happy or sad). |  |  |  |  |  |  |
| **Self-advocacy:**  Increases in the child's ability to communicate their wants and needs with  others (e.g., letting someone know their preferred communication style). |  |  |  |  |  |  |
| **Social skills training:**  Changes in the child's use of certain social skills (e.g., increasing eye contact, initiating conversations in a certain way). |  |  |  |  |  |  |
| **Knowing ways of being social:**  Increasing the child's knowledge of different ways of being social (e.g., some people want eye-contact while other people prefer not to use eye-contact) without the expectation for the child to be social in a certain way. |  |  |  |  |  |  |
| **Focused Interests:**  Decreases in the child's repetitive and focused interests (e.g., increasing range of interests, increasing flexibility in routines). |  |  |  |  |  |  |
| **Sensory behaviours:**  Decreases in the child's sensory seeking (e.g., fidgeting, moving body frequently) and avoiding (e.g., covering ears, avoiding touch) behaviours. |  |  |  |  |  |  |
| If you wish to provide more information about your responses, please enter here. |  |  |  |  |  |  |
| **Social-emotional skills**:  Improvements in the child’s understanding, expression, and regulation of their emotions. |  |  |  |  |  |  |
| **Body movements (also known as motor skills)**:  Improvements in the child's body movements (e.g., use of hands, ability to use objects, walking, running, balance) |  |  |  |  |  |  |
| **School skills:**  Improvements in the child's skills in areas that reflect the education curriculum (e.g., number and letter recognition). |  |  |  |  |  |  |
| **Participation**:  Improvements in skills which help the child take part in daily activities (e.g., increasing their ability to transition between environments, join in with group activities, and attend events within their community). |  |  |  |  |  |  |
| **Self-care skills:**  Improvements in skills that help the child look after themselves in their everyday life (e.g., feeding, getting dressed, toileting). |  |  |  |  |  |  |
| **Play skills:**  Increases in the child's use of neurotypical play skills including using toys for their intended purpose, playing alongside others, and playing  pretend. |  |  |  |  |  |  |
| If you wish to provide more information about your responses, please enter here. |  |  |  |  |  |  |
| **Behaviours that harm self and/or others**:  Decreases in the child's behaviours that harm themselves and others (e.g., hitting, head-banging). |  |  |  |  |  |  |
| **Physical wellbeing:**  Improvements in the child’s physical wellbeing (e.g., their overall health, physical safety). |  |  |  |  |  |  |
| **Mental wellbeing:**  Improvement in the child’s mental wellbeing (e.g., happiness, control over one's life, confidence, time to engage in special interests). |  |  |  |  |  |  |
| **Satisfaction with support:**  What the child thinks about whether the support they are receiving is good or not. |  |  |  |  |  |  |
| If you wish to provide more information about your responses, please enter here. |  |  |  |  |  |  |

**Q3. Outcomes for parents and/or caregivers of young children receiving supports.**

|  | Not an appropriate outcome | An appropriate outcome of support, and a | | | | No opinion/not sure |
| --- | --- | --- | --- | --- | --- | --- |
|  |  | Not at all a priority | Low priority | Medium priority | High Priority |  |
| **Physical wellbeing:**  Improvements in the parent/caregiver’s physical wellbeing (e.g., their overall health, physical safety). |  |  |  |  |  |  |
| **Mental wellbeing:**  Improvement in the parent/caregiver’s mental wellbeing (e.g., confidence, capacity to manage emotions, access to leisure activities). |  |  |  |  |  |  |
| **Knowledge of autism:**  Improvements in the parent/caregiver’s understanding of the characteristics of autism, neurodiversity affirming supports, and how to best support their child. |  |  |  |  |  |  |
| **Parent responsiveness:**  Improvements in how attentive, sensitive, and alert parent/caregivers are to their child's needs, behaviours, and communication attempts. |  |  |  |  |  |  |
| **Parent sense of competence:**  Improvements in how parent/caregiver’s feel about their ability care for their child. |  |  |  |  |  |  |
| **Satisfaction with support**:  What parents/caregivers think is good or not good about the goals, methods, or results of supports their child is receiving. |  |  |  |  |  |  |
| If you wish to provide more information about your responses, please enter here. |  |  |  |  |  |  |

**S5.** Test statistics for analyses conducted.

~~Chi-square analysis of participant perspectives regarding early supports~~

~~Autistic vs. not-autistic: χ2(1) = 13.98,~~ *~~p~~* ~~<.001,~~ *~~V~~* ~~= .281~~

~~Parent vs. not-parent: χ~~^~~2~~^~~(1) = 7.13,~~ *~~p~~* ~~=.008,~~ *~~V~~* ~~= .201~~

~~Practitioner vs. not-practitioner: χ~~^~~2~~^~~(1) = 4.08,~~ *~~p~~* ~~=.043,~~ *~~V~~* ~~= .152.~~

Chi-square analysis of participant perspectives regarding models of disability

Autistic vs. not-autistic: χ^2^(3) = 5.26, *p* = .154, V = .171

Parent vs. not-parent: χ^2^(3) = 6.45, *p* =.092, V = .189

~~Practitioner vs. not-practitioner: χ~~^~~2~~^~~(3) = 18.23, p <.001, V = .318.~~

Chi-square analysis for appropriateness of child outcomes (combined across ages)

Sensory behaviours

Autistic vs. not-autistic: χ^2^(1) = 1.08, *p* = .302, *V* = .088

~~Parent vs. not-parent: χ~~^~~2~~^~~(1) = 8.22,~~ *~~p~~* ~~=.004,~~ *~~V~~* ~~= .244~~

~~Practitioner vs. not-practitioner: χ~~^~~2~~^~~(1) = 12.03,~~ *~~p~~* ~~<.001,~~ *~~V~~* ~~= .295~~

Focused interests

Autistic vs. not-autistic: χ^2^(1) = 3.29, *p* = .070, *V* = .155

~~Parent vs. not-parent: χ~~^~~2~~^~~(1) = 7.75,~~ *~~p~~* ~~=.005,~~ *~~V~~* ~~= .238~~

~~Practitioner vs. not-practitioner: χ2(1) = 11.62,~~ *~~p~~* ~~<.001,~~ *~~V~~* ~~= .291.~~

Social skills training

Autistic vs. not-autistic: χ^2^(1) = 2.50, *p* = .114, *V* = .133

~~Parent vs. not-parent: χ~~^~~2~~^~~(1) = 7.79,~~ *~~p~~* ~~=.005,~~ *~~V~~* ~~= .235~~

~~Practitioner vs. not-practitioner: χ~~^~~2~~^~~(1) = 20.91,~~ *~~p~~* ~~<.001,~~ *~~V~~* ~~= .385.~~

Play skills

Autistic vs. not-autistic: χ^2^(1) = 2.18, *p* = .140, *V* = .127

~~Parent vs. not-parent: χ~~^~~2~~^~~(1) = 16.44,~~ *~~p~~* ~~<.001,~~ *~~V~~* ~~= .349~~

~~Practitioner vs. not-practitioner: χ~~^~~2~~^~~(1) = 20.61,~~ *~~p~~* ~~<.001,~~ *~~V~~* ~~= .391.~~

Knowing ways of being social

Autistic vs. not-autistic: χ^2^(1) = 1.73, *p* = .188, V = .111

Parent vs. not-parent: χ^2^(1) = 0.96, *p* =.327, *V* = .082

Practitioner vs. not-practitioner: χ^2^(1) = 4.08, *p* = .043, *V* = .170.

~~Wilcoxon signed-rank test for change in priority scores for child outcomes (significant results only)~~

~~Understanding of self: z = 3.78,~~ *~~p~~* ~~<.001~~

~~Sensory behaviours: z = 2.81,~~ *~~p~~* ~~=.005~~

~~Social-emotional skills: z = 5.40,~~ *~~p~~* ~~<.001,~~

~~Academic/school skills: z = 3.03,~~ *~~p~~* ~~=.002~~

~~Self-care skills: z = 3.89,~~ *~~p~~* ~~<.001~~

~~Satisfaction with early supports: z = 2.58,~~ *~~p~~* ~~=.010~~

**Table S6.**

*Proportion of Participants Who Indicated Outcomes Were Not an Appropriate Target of Early Supports (Combined)*

|  | All* | Autistic | Non-autistic | Parent | Non-parent | Practitioner | Non-practitioner |
| --- | --- | --- | --- | --- | --- | --- | --- |
|  | *n* (%) | *n* (%) | *n* (%) | *n* (%) | *n* (%) | *n* (%) | *n* (%) |
| Sensory behaviours | 74 (53.6) | 33 (58.9) | 41 (50.0) | 27 (40.9) | 47 (65.3) | 36 (73.5) | 38 (42.7) |
| Focused interests | 74 (54.0) | 36 (63.2) | 38 (47.5) | 27 (41.5) | 47 (65.3) | 36 (73.5) | 38 (43.2) |
| Social skills training | 69 (48.9) | 32 (57.1) | 37 (43.5) | 25 (36.8) | 44 (60.3) | 38 (74.5) | 31 (34.4) |
| Play skills** | 67 (49.6) | 31 (57.4) | 36 (44.4) | 20 (31.3) | 47 (66.2) | 37 (75.5) | 30 (34.9) |
| Knowing ways of being social | 13 (9.2) | 3 (5.3) | 10 (11.8) | 8 (11.6) | 5 (6.8) | 8 (15.7) | 5 (5.5) |
| Early school skills | 8 (5.9) | 3 (5.8) | 5 (6.0) | 5 (7.7) | 3 (4.2) | 3 (6.0) | 5 (5.8) |
| Cognition | 5 (3.6) | 2 (3.6) | 3 (3.6) | 0 | 5 (7.0) | 4 (8.3) | 1 (1.1) |
| Participation | 5 (3.7) | 4 (7.8) | 1 (1.2) | 1 (1.6) | 4 (5.6) | 3 (6.0) | 2 (2.4) |
| Behaviours that harm self and/or others | 4 (3.0) | 3 (5.7) | 1 (1.3) | 3 (4.9) | 1 (1.4) | 2 (4.0) | 2 (2.4) |
| Satisfaction with supports | 4 (3.0) | 2 (3.6) | 2 (2.5) | 2 (3.1) | 2 (2.8) | 0 | 4 (4.6) |
| Motor skills | 4 (3.1) | 2 (4.0) | 2 (2.5) | 0 | 4 (5.8) | 2 (4.3) | 2 (2.4) |
| Receptive language | 3 (2.2) | 1 (1.8) | 2 (2.4) | 0 | 3 (4.2) | 2 (4.1) | 1 (1.1) |
| Expressive language | 2 (1.5) | 0 | 2 (2.4) | 0 | 2 (2.8) | 2 (4.0) | 0 |
| Social emotional skills | 2 (1.5) | 0 | 2 (2.4) | 1 (1.6) | 1 (1.4) | 1 (2.0) | 1 (1.2) |
| Inclusivity and accessibility of environments | 1 (0.7) | 1 (1.7) | 0 | 0 | 1 (1.4) | 0 | 1 (1.1) |
| Self-care skills | 1 (0.7) | 1 (1.9) | 0 | 0 | 1 (1.4) | 0 | 1 (1.2) |
| Physical wellbeing | 1 (0.7) | 1 (1.9) | 0 | 1 (1.5) | 0 | 1 (2.0) | 0 |
| Mental wellbeing | 1 (0.7) | 0 | 1 (1.2) | 1 (1.5) | 0 | 0 | 1 (1.1) |
| Sensory tools | 0 | 0 | 0 | 0 | 0 | 0 | 0 |
| Understanding self | 0 | 0 | 0 | 0 | 0 | 0 | 0 |
| Self advocacy | 0 | 0 | 0 | 0 | 0 | 0 | 0 |

**Table S7.**

*Priority Ratings by Group of Targeted Outcomes for Autistic Children Aged 0-5 Years*

|  | All | | Autistic | | | Non-autistic | | | Parent | | | Non-parent | | | Practitioner | | | Non-practitioner | | |
| --- | --- | --- | --- | --- | --- | --- | --- | --- | --- | --- | --- | --- | --- | --- | --- | --- | --- | --- | --- | --- |
| Outcomes | *n* | *Mean*  *(SD)* | *n* | *Mean*  *(SD)* | Rank | *n* | *Mean*  *(SD)* | Rank | *n* | *Mean*  *(SD*) | Rank | *n* | *Mean*  *(SD)* | Rank | *n* | *Mean*  *(SD)* | Rank | *n* | *Mean*  *(SD)* | Rank |
| Mental wellbeing | 153 | 3.73  (0.70) | 62 | 3.68  (0.74) | 1 | 91 | 3.77  (0.67) | 1 | 72 | 3.76  (0.68) | 1 | 81 | 3.70  (0.71) | 1 | 55 | 3.85  (0.52) | 1 | 98 | 3.66  (0.77) | 1 |
| Behaviours that harm self and/or others | 149 | 3.54  (0.93) | 60 | 3.45  (1.03) | 3 | 89 | 3.60  (0.85) | 2 | 69 | 3.48  (0.98) | 3 | 80 | 3.59  (0.88) | 3 | 54 | 3.57  (0.92) | 6 | 95 | 3.52  (0.93) | 2 |
| Physical wellbeing | 153 | 3.52  (0.90) | 61 | 3.46  (0.98) | 2 | 92 | 3.55  (0.86) | 4 | 72 | 3.42  (0.96) | 4 | 81 | 3.60  (0.85) | 2 | 55 | 3.69  (0.77) | 4 | 98 | 3.42  (0.96) | 4 |
| Inclusivity and accessibility | 170 | 3.51  (0.89) | 68 | 3.41  (0.97) | 6 | 102 | 3.57  (0.83) | 3 | 80 | 3.63  (0.79) | 2 | 90 | 3.40  (0.96) | 7 | 63 | 3.75  (0.67) | 2 | 107 | 3.36  (0.97) | 5 |
| Understanding self | 164 | 3.43  (0.93) | 58 | 3.43  (0.88) | 4 | 97 | 3.38  (0.96) | 6 | 77 | 3.42  (0.94) | 5 | 87 | 3.44  (0.92) | 6 | 60 | 3.28  (0.99) | 7 | 104 | 3.51  (0.88) | 3 |
| Sensory tools | 169 | 3.38  (0.96) | 68 | 3.38  (0.98) | 7 | 101 | 3.38  (0.95) | 7 | 80 | 3.26  (0.99) | 7 | 89 | 3.48  (0.92) | 4 | 62 | 3.71  (0.71) | 3 | 107 | 3.19  (1.03) | 7 |
| Satisfaction with supports | 149 | 3.34  (1.00) | 62 | 3.42  (0.97) | 5 | 87 | 3.28  (1.02) | 8 | 70 | 3.19  (1.05) | 8 | 79 | 3.47  (0.93) | 5 | 52 | 3.65  (0.76) | 5 | 97 | 3.16  (1.07) | 8 |
| Expressive language | 161 | 3.27  (1.03) | 63 | 3.08  (1.10) | 8 | 98 | 3.40  (0.97) | 5 | 74 | 3.38  (0.98) | 6 | 87 | 3.18  (1.07) | 8 | 62 | 3.24  (1.05) | 8 | 99 | 3.29  (1.02) | 6 |
| Receptive language | 162 | 3.04  (1.07) | 64 | 2.84  (1.09) | 11 | 98 | 3.16  (1.05) | 9 | 75 | 3.16  (1.05) | 9 | 87 | 2.93  (1.09) | 10 | 61 | 2.93  (1.08) | 11 | 101 | 3.10  (1.07) | 10 |
| Self-care skills | 150 | 3.01  (1.06) | 58 | 2.93  (1.11) | 9 | 92 | 3.07  (1.04) | 11 | 71 | 3.06  (1.03) | 12 | 79 | 2.97  (1.10) | 9 | 54 | 2.94  (1.09) | 10 | 96 | 3.05  (1.05) | 11 |
| Social emotional skills | 152 | 3.01  (1.07) | 58 | 2.76  (1.08) | 12 | 94 | 3.16  (1.04) | 10 | 72 | 3.15  (1.10) | 10 | 80 | 2.88  (1.04) | 11 | 55 | 2.95  (1.06) | 9 | 97 | 3.04  (1.08) | 12 |
| Cognition | 161 | 2.87  (1.12) | 64 | 2.88  (1.13) | 10 | 97 | 2.87  (1.11) | 13 | 77 | 3.06  (1.08) | 11 | 84 | 2.69  (1.13) | 13 | 58 | 2.36  (0.99) | 14 | 103 | 3.16  (1.09) | 9 |
| Participation | 152 | 2.84  (1.07) | 59 | 2.56  (1.06) | 14 | 93 | 3.02  (1.05) | 12 | 71 | 2.97  (1.07) | 13 | 81 | 2.73  (1.06) | 12 | 55 | 2.73  (1.08) | 12 | 97 | 2.91  (1.06) | 13 |
| Motor skills | 144 | 2.68  (1.04) | 56 | 2.45  (0.93) | 15 | 88 | 2.83  (1.07) | 14 | 67 | 2.84  (1.04) | 14 | 77 | 2.55  (1.02) | 14 | 51 | 2.49  (0.97) | 13 | 93 | 2.78  (1.06) | 14 |
| Knowing ways of being social | 163 | 2.44  (1.15) | 67 | 2.66  (1.20) | 13 | 96 | 2.29  (1.09) | 15 | 76 | 2.34  (1.21) | 15 | 87 | 2.53  (1.10) | 15 | 61 | 2.15  (1.01) | 15 | 102 | 2.62  (1.19) | 15 |
| Early school skills | 153 | 2.16  (0.96) | 60 | 2.18  (1.00) | 16 | 93 | 2.15  (0.93) | 16 | 73 | 2.23  (0.98) | 16 | 80 | 2.10  (0.94) | 16 | 55 | 1.89  (0.74) | 16 | 98 | 2.32  (1.03) | 16 |
| Social skills training | 162 | 1.77  (1.09) | 66 | 1.68  (1.07) | 17 | 96 | 1.83  (1.10) | 18 | 71 | 2.06  (1.16) | 17 | 81 | 1.49  (0.97) | 18 | 55 | 1.44  (0.52) | 17 | 97 | 1.94  (1.20) | 18 |
| Play | 152 | 1.76  (1.05) | 61 | 1.59  (0.97) | 18 | 91 | 1.87  (1.09) | 17 | 75 | 2.03  (1.15) | 18 | 87 | 1.55  (0.88) | 17 | 61 | 1.21  (0.90) | 19 | 101 | 2.11  (1.09) | 17 |
| Focused interests | 159 | 1.53  (0.86) | 67 | 1.37  (0.71) | 19 | 92 | 1.64  (0.94) | 19 | 73 | 1.70  (0.98) | 19 | 86 | 1.38  (0.72) | 19 | 59 | 1.22  (0.53) | 18 | 100 | 1.71  (0.97) | 19 |
| Sensory behaviours | 160 | 1.38  (0.76) | 65 | 1.22  (0.52) | 20 | 95 | 1.49  (0.87) | 20 | 74 | 1.57  (0.91) | 20 | 86 | 1.22  (0.56) | 20 | 61 | 1.10  (0.44) | 20 | 99 | 1.56  (0.86) | 20 |

**Table S8.**

*Priority Ratings by Group of Targeted Outcomes for Autistic Children Aged 6-12 Years*

|  | All | | Autistic | | | Non-autistic | | | Parent | | | Non-parent | | | Practitioner | | | Non-practitioner | | |
| --- | --- | --- | --- | --- | --- | --- | --- | --- | --- | --- | --- | --- | --- | --- | --- | --- | --- | --- | --- | --- |
|  | *n* | *Mean*  *(SD)* | *n* | *Mean*  *(SD)* | Rank | *n* | *Mean*  *(SD)* | Rank | *n* | *Mean*  *(SD)* | Rank | *n* | *Mean*  *(SD)* | Rank | *n* | *Mean*  *(SD)* | Rank | *n* | *Mean*  *(SD)* | Rank |
| Mental wellbeing | 139 | 3.84  (0.54) | 55 | 3.82  (0.58) | 1 | 84 | 3.86  (0.52) | 1 | 66 | 3.82  (0.58) | 1 | 73 | 3.86  (0.51) | 1 | 51 | 3.96  (0.28) | 1 | 88 | 3.77  (0.64) | 1 |
| Understanding self | 142 | 3.72  (0.70) | 57 | 3.72  (0.70) | 2 | 85 | 3.72  (0.70) | 3 | 69 | 3.71  (0.71) | 2 | 73 | 3.73  (0.69) | 3 | 51 | 3.88  (0.48) | 3 | 91 | 3.63  (0.78) | 3 |
| Self-advocacy skills | 142 | 3.70  (0.73) | 57 | 3.67  (0.79) | 3 | 85 | 3.72  (0.70) | 4 | 69 | 3.71  (0.71) | 3 | 73 | 3.68  (0.76) | 4 | 51 | 3.88  (0.48) | 4 | 91 | 3.59  (0.83) | 4 |
| Behaviours that harm self and/or others | 133 | 3.68  (0.81) | 53 | 3.53  (1.01) | 4 | 80 | 3.78  (0.64) | 2 | 61 | 3.61  (0.86) | 6 | 72 | 3.74  (0.77) | 2 | 50 | 3.70  (0.84) | 8 | 83 | 3.66  (0.80) | 2 |
| Physical wellbeing | 138 | 3.59  (0.83) | 54 | 3.43  (0.94) | 8 | 84 | 3.69  (0.73) | 6 | 65 | 3.52  (0.89) | 7 | 73 | 3.64  (0.77) | 5 | 51 | 3.75  (0.72) | 7 | 87 | 3.49  (0.87) | 5 |
| Inclusivity and accessibility | 144 | 3.58  (0.84) | 58 | 3.45  (0.96) | 6 | 86 | 3.67  (0.74) | 7 | 70 | 3.66  (0.76) | 4 | 74 | 3.51  (0.91) | 8 | 51 | 3.88  (0.48) | 2 | 93 | 3.42  (0.95) | 7 |
| Sensory tools | 142 | 3.51  (0.89) | 58 | 3.43  (0.94) | 7 | 84 | 3.56  (0.86) | 8 | 69 | 3.43  (0.93) | 8 | 73 | 3.58  (0.85) | 7 | 50 | 3.84  (0.55) | 5 | 92 | 3.33  (0.98) | 8 |
| Satisfaction with supports | 139 | 3.50  (0.94) | 55 | 3.53  (0.98) | 5 | 84 | 3.48  (0.92) | 9 | 66 | 3.38  (1.00) | 10 | 73 | 3.60  (0.88) | 6 | 51 | 3.80  (0.60) | 6 | 88 | 3.32  (1.06) | 9 |
| Social emotional skills | 136 | 3.49  (0.89) | 52 | 3.15  (1.00) | 9 | 84 | 3.70  (0.74) | 5 | 64 | 3.66  (0.76) | 5 | 72 | 3.35  (0.97) | 9 | 50 | 3.50  (0.91) | 9 | 86 | 3.49  (0.88) | 6 |
| Self-care skills | 136 | 3.27  (1.00) | 52 | 3.10  (1.09) | 10 | 84 | 3.38  (0.93) | 11 | 64 | 3.31  (0.96) | 11 | 72 | 3.24  (1.04) | 10 | 50 | 3.32  (0.96) | 10 | 86 | 3.24  (1.03) | 10 |
| Expressive language | 140 | 3.25  (1.03) | 55 | 2.93  (1.09) | 11 | 85 | 3.46  (0.93) | 10 | 68 | 3.40  (0.95) | 9 | 72 | 3.11  (1.08) | 11 | 50 | 3.28  (1.03) | 11 | 90 | 3.23  (1.03) | 11 |
| Receptive language | 141 | 2.99  (1.05) | 56 | 2.71  (1.02) | 13 | 85 | 3.18  (1.03) | 12 | 68 | 3.18  (0.99) | 12 | 73 | 2.82  (1.07) | 13 | 50 | 2.86  (1.09) | 13 | 91 | 3.07  (1.02) | 13 |
| Participation | 135 | 2.96  (1.08) | 51 | 2.65  (1.09) | 15 | 84 | 3.14  (1.03) | 13 | 64 | 3.08  (1.07) | 14 | 71 | 2.85  (1.08) | 12 | 50 | 2.90  (1.09) | 12 | 85 | 2.99  (1.07) | 14 |
| Cognition | 142 | 2.93  (1.04) | 56 | 2.89  (1.06) | 12 | 86 | 2.95  (1.04) | 14 | 69 | 3.13  (1.00) | 13 | 73 | 2.74  (1.05) | 14 | 50 | 2.50  (0.97) | 16 | 92 | 3.16  (1.01) | 12 |
| Knowing ways of being social | 143 | 2.72  (1.10) | 57 | 2.68  (1.09) | 14 | 86 | 2.74  (1.11) | 16 | 70 | 2.74  (1.10) | 16 | 73 | 2.70  (1.10) | 15 | 51 | 2.51  (1.12) | 15 | 92 | 2.84  (1.07) | 15 |
| Motor skills | 136 | 2.70  (1.04) | 53 | 2.42  (0.95) | 16 | 83 | 2.88  (1.05) | 15 | 65 | 2.89  (1.05) | 15 | 71 | 2.52  (1.00) | 16 | 49 | 2.57  (0.98) | 14 | 87 | 2.77  (1.06) | 16 |
| Academic Skills | 136 | 2.50  (1.03) | 52 | 2.37  (0.95) | 17 | 84 | 2.58  (1.08) | 17 | 65 | 2.65  (1.08) | 17 | 71 | 2.37  (0.97) | 17 | 50 | 2.26  (0.94) | 17 | 86 | 2.64  (1.06) | 17 |
| Social skills training | 141 | 1.95  (1.19) | 56 | 1.79  (1.12) | 18 | 85 | 2.06  (1.23) | 18 | 68 | 2.29  (1.26) | 18 | 73 | 1.63  (1.03) | 18 | 51 | 1.35  (0.84) | 18 | 90 | 2.29  (1.23) | 18 |
| Play | 136 | 1.84  (1.10) | 54 | 1.67  (0.95) | 19 | 82 | 1.95  (1.17) | 19 | 65 | 2.12  (1.17) | 19 | 71 | 1.58  (0.97) | 19 | 49 | 1.35  (0.78) | 19 | 87 | 2.11  (1.16) | 19 |
| Focused interests | 139 | 1.69  (0.98) | 57 | 1.51  (0.89) | 20 | 82 | 1.82  (1.02) | 20 | 67 | 1.93  (0.99) | 20 | 72 | 1.47  (0.92) | 20 | 49 | 1.29  (0.68) | 20 | 90 | 1.91  (1.05) | 20 |
| Sensory behaviours | 139 | 1.62  (0.99) | 56 | 1.39  (0.68) | 21 | 83 | 1.77  (1.13) | 21 | 66 | 1.92  (1.13) | 21 | 73 | 1.34  (0.75) | 21 | 50 | 1.22  (0.65) | 21 | 89 | 1.84  (1.08) | 21 |

**Table S9.**

*Linear Regression Predicting Mean Priority Scores of Child Outcomes*

|  | Inclusivity and accessibility | | Sensory  tools | | Cognition | | Receptive  language | | Expressive  language | | Social  Skills training | |
| --- | --- | --- | --- | --- | --- | --- | --- | --- | --- | --- | --- | --- |
| Predictors | β | *p* | β | *p* | β | *p* | β | *p* | β | *p* | β | *p* |
| Role/Identity |  |  |  |  |  |  |  |  |  |  |  |  |
| Autistic vs. not | .01 | .933 | .15 | .353 | -.11 | .530 | -.34 | .066 | -.31 | .103 | -.24 | .176 |
| Parent vs. not | .59 | <.001 | .16 | .315 | -.09 | .626 | .00 | .990 | -.02 | .917 | -.00 | .997 |
| Practitioner vs. not | .55 | .001 | .68 | <.001 | -.66 | .001* | -.16 | .428 | -.06 | .769 | -.77 | <.001 |
| Perspectives on early supports |  |  |  |  |  |  |  |  |  |  |  |  |
| It depends vs. appropriate | .25 | .056 | -.08 | .567 | -.40 | .012 | -.42 | .011 | -.33 | .047 | -.77 | <.001 |
| Model of disability |  |  |  |  |  |  |  |  |  |  |  |  |
| Biopsychosocial vs. social | -.57 | .003 | -.34 | .108 | .35 | .132 | .42 | .081 | .15 | .523 | .20 | .360 |
| Neurodiversity vs. social | -.30 | .090 | .05 | .793 | .39 | .079 | .30 | .197 | -.03 | .912 | -.31 | .152 |
| Other vs. social | -.53 | .037 | -.31 | .273 | .05 | .869 | .36 | .266 | .34 | .294 | .62 | .045 |

Inclusivity and accessibility: *F*(7, 158) = 5.60, *p* <.001, R^2^=.199

Sensory tools: *F*(7, 157) = 3.39, *p* =.002, R^2^=.131

Cognition: *F*(7, 152) = 5.40, *p* <.001, R^2^=.199

Receptive language: *F*(7, 153) = 3.45, *p* =.002, R^2^=.136

Expressive language: *F*(7, 152) = 2.04, *p* =.053, R^2^=.086

Social skills training: *F*(7, 150) = 11.77, *p* <.001, R^2^=.354

**Table S9 cont.**

|  | Knowing ways of being social | | Focused interests | | Motor skills | | Participation | | Play | | Physical wellbeing | |
| --- | --- | --- | --- | --- | --- | --- | --- | --- | --- | --- | --- | --- |
| Predictors | β | *p* | β | *p* | β | *p* | β | *p* | β | *p* | β | *p* |
| Role/Identity |  |  |  |  |  |  |  |  |  |  |  |  |
| Autistic vs. not | .05 | .803 | -.33 | .030 | -.48 | .011 | -.43 | .030 | -.15 | .412 | -.24 | .133 |
| Parent vs. not | -.31 | .089 | -.04 | .778 | .06 | .768 | -.06 | .765 | .13 | .483 | -.06 | .705 |
| Practitioner vs. not | -.32 | .121 | -.55 | .002 | -.18 | .390 | -.09 | .695 | -.45 | .031 | .40 | .026 |
| Perspectives on early supports |  |  |  |  |  |  |  |  |  |  |  |  |
| It depends vs. appropriate | -.44 | .007 | -.47 | <.001 | -.36 | .028 | -.54 | .002 | -.70 | <.001 | .28 | .042 |
| Model of disability |  |  |  |  |  |  |  |  |  |  |  |  |
| Biopsychosocial vs. social | -.04 | .864 | .34 | .077 | -.27 | .257 | .04 | .869 | .09 | .692 | .16 | .431 |
| Neurodiversity vs. social | .41 | .068 | .27 | .147 | .21 | .378 | .28 | .247 | .13 | .560 | .66 | <.001 |
| Other vs. social | .46 | .150 | .55 | .036 | .19 | .540 | .30 | .378 | .52 | .097 | .65 | .014 |

Knowing ways of being social: *F*(7, 152) = 3.67, *p* <.001, R^2^=.145

Focused interest: *F*(7, 149) = 8.03, *p* <.001, R^2^=.274

Motor skills: *F*(7, 139) = 3.82, *p* <.001, R^2^=.161

Participation: *F*(7, 141) = 3.61, *p* <.001, R^2^=.152

Play: *F*(7, 142) = 7.04, *p* <.001, R^2^=.258

Physical wellbeing: *F*(7, 142) = 4.12, *p* <.001, R^2^=.169

**Table S9 cont.**

|  | Understanding self  0-5 years | | Sensory behaviours  0-5 years | | Sensory behaviours  6-12 years | | Social emotional skills  0-5 years | | Satisfaction with supports 0-5 years | |
| --- | --- | --- | --- | --- | --- | --- | --- | --- | --- | --- |
| Predictors | β | *p* | β | *p* | β | *p* | β | *p* | β | *p* |
| Role/Identity |  |  |  |  |  |  |  |  |  |  |
| Autistic vs. not | -.12 | .484 | -.31 | .026 | -.27 | .136 | -.32 | .146 | .05 | .795 |
| Parent vs. not | -.07 | .668 | -.04 | .743 | .12 | .503 | .08 | .722 | .11 | .541 |
| Practitioner vs. not | -.21 | .284 | -.48 | .002* | -.38 | .063 | .08 | .754 | .54 | .014 |
| Perspectives on early supports |  |  |  |  |  |  |  |  |  |  |
| It depends vs. appropriate | .41 | .010 | -.35 | .005* | -.62 | <.001 | -.35 | .060 | .53 | .002 |
| Model of disability |  |  |  |  |  |  |  |  |  |  |
| Biopsychosocial vs. social | .32 | .151 | .21 | .225 | .17 | .452 | .13 | .632 | -.63 | .008 |
| Neurodiversity vs. social | .61 | .005 | -.19 | .251 | .40 | .071 | .52 | .055 | .04 | .856 |
| Other vs. social | .38 | .218 | .37 | .124 | .69 | .027 | .56 | .125 | .11 | .711 |

Understanding self, 0-5 years: *F*(7, 152) = 2.63, *p* =.014, R^2^=.108

Sensory behaviours, 0-5 years: *F*(8, 141) = 6.52, *p* <.001, R^2^=.270

Sensory behaviours, 6-12 years: *F*(7, 129) = 7.28, *p* <.001, R^2^=.283

Social emotional skills, 0-5 years: *F*(7, 141) = 2.54, *p* = .027, R^2^=.105

Satisfaction with supports, 0-5 years: *F*(7, 139) = 5.83, *p* < .001, R^2^=.227

**Table S10.**

*Priority Ratings for Parent Outcomes*

|  | All | | Autistic | | | Non-autistic | | | Parent | | | Non-parent | | | Practitioner | | | Non-practitioner | | |
| --- | --- | --- | --- | --- | --- | --- | --- | --- | --- | --- | --- | --- | --- | --- | --- | --- | --- | --- | --- | --- |
|  | N | Mean  (SD) | n | Mean  (SD) | Rank | n | Mean  (SD) | Rank | n | Mean  (SD) | Rank | n | Mean  (SD) | Rank | n | Mean  (SD) | Rank | n | Mean  (SD) | Rank |
| Knowledge of autism | 139 | 3.78  0.62 | 54 | 3.81  0.59 | 1 | 85 | 3.76  0.65 | 1 | 67 | 3.67  0.75 | 1 | 72 | 3.89  0.46 | 1 | 51 | 3.92  0.39 | 1 | 88 | 3.70  0.71 | 1 |
| Parent responsiveness | 138 | 3.61  0.82 | 54 | 3.59  0.81 | 2 | 84 | 3.62  0.83 | 3 | 66 | 3.58  0.88 | 3 | 72 | 3.64  0.77 | 2 | 51 | 3.80  0.60 | 2 | 87 | 3.49  0.91 | 2 |
| Parent mental wellbeing | 136 | 3.55  0.85 | 52 | 3.33  0.98 | 3 | 84 | 3.69  0.73 | 2 | 67 | 3.61  0.80 | 2 | 69 | 3.49  0.90 | 4 | 49 | 3.71  0.71 | 4 | 87 | 3.46  0.91 | 3 |
| Parent sense of competence | 138 | 3.41  0.95 | 54 | 3.26  1.03 | 4 | 84 | 3.51  0.88 | 5 | 66 | 3.30  1.01 | 5 | 72 | 3.51  0.89 | 3 | 51 | 3.69  0.73 | 5 | 87 | 3.25  1.03 | 4 |
| Parent satisfaction with supports | 137 | 3.41  0.95 | 53 | 3.11  1.09 | 5 | 84 | 3.60  0.81 | 4 | 66 | 3.38  0.96 | 4 | 71 | 3.44  0.95 | 5 | 50 | 3.76  0.66 | 3 | 87 | 3.21  1.04 | 5 |
| Parent physical wellbeing | 135 | 3.26  1.01 | 52 | 3.10  1.09 | 6 | 83 | 3.36  0.96 | 6 | 67 | 3.30  0.98 | 6 | 68 | 3.22  1.05 | 6 | 48 | 3.42  0.92 | 6 | 87 | 3.17  1.06 | 6 |

**Table S11.**

*Linear Regression Predicting Mean Priority Scores of Parent Outcomes*

|  | Sense of competence | | Satisfaction with supports | | Physical wellbeing | |
| --- | --- | --- | --- | --- | --- | --- |
| Predictors | β | *p* | β | *p* | β | *p* |
| Role/Identity |  |  |  |  |  |  |
| Autistic vs. not | -.35 | .075 | -.34 | .087 | -.13 | .565 |
| Parent vs. not | -.10 | .613 | .09 | .652 | .16 | .458 |
| Practitioner vs. not | .43 | .056 | .61 | .008 | .53 | .038 |
| Perspectives on early supports |  |  |  |  |  |  |
| It depends vs. appropriate | .28 | .108 | .03 | .873 | -.01 | .981 |
| Model of disability |  |  |  |  |  |  |
| Biopsychosocial vs. social | -.22 | .382 | -.15 | .548 | .20 | .474 |
| Neurodiversity vs. social | .24 | .321 | .20 | .410 | .54 | .049 |
| Other vs. social | .24 | .462 | .28 | .397 | .61 | .102 |

Sense of competence: *F*(7, 128) = 2.28, *p* = .010, R^2^=.132

Satisfaction with supports: *F*(7, 127) = 2.33, *p* = .010, R^2^=.133

Physical wellbeing: *F*(7, 125) = 1.36, *p* = .232, R^2^=.070
